# Relative frequency dynamics and loading of beet necrotic yellow vein virus genomic RNAs during the acquisition by its vector *Polymyxa betae*

**DOI:** 10.1128/jvi.01410-24

**Published:** 2024-12-16

**Authors:** Yi Guo, Mattia Dall'Ara, David Baldo, David Gilmer, Claudio Ratti

**Affiliations:** 1 DISTAL-Plant Pathology, University of Bologna 304050 , Bologna, Italy; 2 Institute for Sustainable Plant Protection, National Research Council of Italy 111214 , Turin, Italy; 3 Ri.NOVA Società Cooperativa, Cesena, Italy; 4 Institut de biologie moléculaire des plantes, CNRS, Université de Strasbourg 27083 , Strasbourg, France; Iowa State University, Ames, Iowa, USA

**Keywords:** multipartite virus, *Benyvirus*, BNYVV, *Polymyxa betae*, genome formula

## Abstract

The beet necrotic yellow vein virus (BNYVV) is a multipartite virus with the highest number (up to five) of genomic segments among RNA viruses. Classified as a soil-borne virus, it is persistently transmitted by the protozoan *Polymyxa betae*. Previous studies have demonstrated that the relative frequency of the BNYVV genomic RNAs was modified depending on the host plant as well as the infected organ, resulting in distinct stoichiometric ratios between the viral RNAs. In this study, we investigate whether infection by the vector *P. betae* influences the relative abundance of BNYVV RNAs within the roots of the host plant *Beta vulgaris*. Furthermore, we examine the relative frequency of BNYVV genomic segments and the viral load of BNYVV at two different stages of *P. betae*’s biological cycle: zoospore and resting spore. Our finding offers new insights into understanding the biology of this soil-borne virus and its vector. Notably, the variations in the relative accumulation of BNYVV RNAs observed in zoospores and resting spores, along with a higher viral load in zoospores compared to resting spores, invite consideration of the virus’s replicative capacity within the vector.

**IMPORTANCE:**

Our understanding of the transmission of plant viruses by protozoan vectors remains poor and fragmented. The fate of viral elements in the living stages of the vector is unknown. Here, we first established a protocol allowing the purification of two forms of the vector free of cellular contaminants. This permitted the examination of the relative frequencies of beet necrotic yellow vein virus RNAs in the roots of its natural host and in two forms of its protozoan vector, *Polymyxa betae*, responsible for virus transmission. Our findings provide new insights into virus behavior during vector transmission, allowing us to analyze how the virus regulates its RNA frequencies and load within the vector. By focusing on the early stages of viral transmission and separating virus acquisition from transmission to new hosts, we pave the way for experiments aimed at elucidating the molecular mechanisms behind viral acquisition and the maintenance of viral genome integrity by *P. betae*.

## INTRODUCTION

Viruses employ various ways to organize and regulate the expression of their genetic material, such as segmenting the genome into distinct genomic elements. While segmented viruses maintain genomic integrity by packaging a complete set of genomic segments within a single viral particle, multipartite viruses keep genomic entities separate even after encapsidation, packaging them into independent viral particles (1). Despite the primary cost of losing genomic components during transmission within and between hosts (2), multipartite genomic organization is widespread among plant and fungal viruses, rare in animals, and absent in bacterial and archaeal viruses (3, 4).

Evolutionary virologists are intrigued by this biological system and its potential advantages over monopartite systems, but debate persists due to limited empirical evidence (5). A recent study, based on a mathematical model, suggests that viral genome segmentation might have emerged from defective genomes, referred to as “cheats,” into populations of non-segmented ones. These cheats benefit from full-length genomes to compensate for their missing parts and usually possess significant fitness advantages over wild-type counterparts leading to a potential formation of genome populations solely consisting of mutually complementary cheats (6). The analytical model suggests that the transition can still occur in multipartite viruses with realistic levels of cheat co-infection (7).

Other advantages, listed below, are traditionally associated with segmented and multipartite viruses. However, there are no clear explanations for why multipartitism is more common among phytoviruses and mycoviruses than among viruses infecting other hosts. Genomic segments provide means to enhance viral fitness (8), increase the replication rates of individual segments (9), reduce the occurrence of deleterious mutations (10), and allow a fine-tuned regulation of gene expression by modulating the relative frequencies of the different segments during infection. These frequencies reach an equilibrium, establishing specific stoichiometric relationships between the viral genomic elements, known as setpoint genome formulas (SGFs) (11), which vary depending on the infected host (11–19) and for some viruses even on the type of infected tissue (18). In general, gene copy number variation strongly impacts the gene expression and phenotype of the organisms (20), thereby adjusting their gene copy number (CN) to respond to new conditions (21–26). However, while gene expression modulation based on relative genomic element frequencies has been demonstrated in viruses such as the faba bean necrotic stunt virus (FBNSV), a multipartite DNA virus with eight genomic segments (27), to date, achieving specific SGFs in a specific infectious context has not been associated with clear benefits.

The investigation into the genome formula modulation has been extended to the multipartite virus transmission, FBNSV, three bipartite begomoviruses, and the tenuivirus rice stripe virus (RSV), the sole RNA virus in the list, all establish characteristic SGFs in the vector different from the host (15, 17, 28), even in the absence of replication in the vector (29), except for the squash leaf curl China virus (16). In laboratory conditions, aphids can gradually rebuild the complete set of genomic elements within their bodies at various feeding times, while multiple insects can acquire partial genomic sets and then alternatively reconstruct them into a complete one after transmission to a new host (30). This indicates that virus acquisition by the vector is not dependent on absorbing collective infection units (31) formed in the host, characterized by a host-independent SGF.

In this context, our research raises the question of whether the genome formula of the soil-borne beet necrotic yellow vein virus (BNYVV) changes after its acquisition by the vector *Polymyxa betae* by analyzing two stages of the protist cycle. BNYVV is a positive single-stranded RNA multipartite virus, classified within the *Benyvirus* genus of the *Benyviridae* family (32), and it is the causative agent of rhizomania disease in *Beta vulgaris* plants (33). Depending on the genotype, its genome can be tetrapartite or pentapartite (34). RNA1 and -2 are essential segments, necessary and sufficient to establish infection when mechanically inoculated into species of *Chenopodium*, *Spinacia*, and *Nicotiana* (32). In contrast, RNA3, -4, and -5 are involved in transmission, pathogenicity, and systemic movement within *Beta* hosts (32). RNA1 encodes the viral replicase, while RNA2 encodes the major and minor structural proteins, proteins facilitating cell-to-cell movement, and an RNA silencing suppressor (32). RNA3 expresses the pathogenicity determinant and contributes to long-distance movement in *Beta* hosts (35, 36), while RNA4 is critical for efficient vector transmission and silencing suppression in the host’s root system (37). When present, RNA5 exacerbates host symptoms (38).

As a soil-borne virus, BNYVV relies on the vector *P. betae* for transmission between plants, and the obligate intracellular nature of the parasite involves a different virus acquisition process compared to arthropod-mediated transmission. This process requires cytoplasmic sharing between one infected root hair or epidermal root cell and the protozoan, resulting in the formation of a multinucleated and viruliferous plasmodium (39, 40). Subsequently, this plasmodium matures into either a zoosporangium for immediate virus dissemination in the soil to new hosts through biflagellate secondary zoospores or into a cystosorus for resting spore formation (39, 40). In the latter case, the virus can persist for years in the soil until the resting spores germinate into new primary zoospores and release their cytoplasmic contents after encysting into the lateral root cell of the host, thus establishing infection (39, 40). Despite its importance, it is unclear if the virus can replicate in the vector, contributing to its circulative transmission. Although viral replication products have been identified in resting spores (41), their temporal quantitative increase remains unexplored post-spore isolation, raising the possibility of their simple acquisition from the host cytoplasm during plasmodium formation.

For the first time, we isolated and purified *P. betae* zoospores and resting spores viruliferous for the genotype B of BNYVV, which comprise RNA1, RNA2, RNA3, and RNA4 genomic RNAs (34). Our aim was to quantify the viral genomic content using reverse transcriptase digital droplet PCR (RT-ddPCR). This approach allowed us to examine how the relative frequencies of BNYVV genomic RNAs change during the virus’s acquisition from infected root tissues of *B. vulgaris* to the extracellular forms of the vector, while also evaluating the viral load for each form. Our study offers novel insights into the virus biology within a system involving both the host and vector.

## RESULTS

### Defining BNYVV SGF in roots of *B. vulgaris* with or without *P. betae*

**Fig 1:**
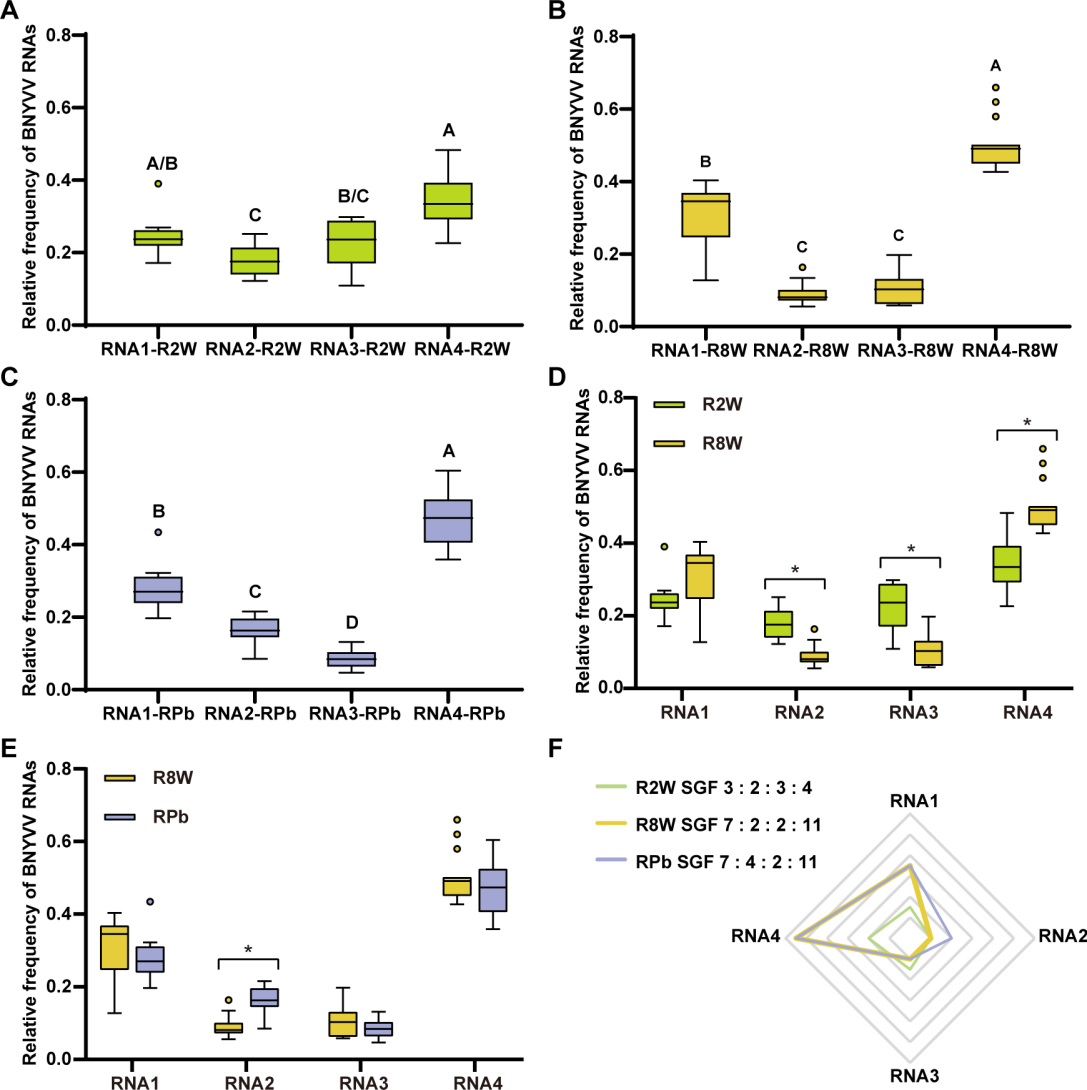
BNYVV vRNAs relative accumulation in *B. vulgaris* roots. (**A and B**) BNYVV vRNAs relative accumulation in *B. vulgaris* roots infected with BNYVV for 2 w.p.i. (R2W) and 8 w.p.i. (R8W). (**C**) BNYVV vRNAs relative accumulation in *B. vulgaris* roots infected with BNYVV and *P. betae* (RPb). Each boxplot represents the middle 50% of the subgroup population. The line through the box represents the median, and the lines extending from the box represent the upper and lower 25% of the data (excluding outliers). Outliers are represented by small circles (°). Letters indicate significant differences between dependent variables within each group, which were estimated by Wilcoxon signed-rank test. The *P*-values from Wilcoxon signed-rank test are shown in Table S3. (**D**) Pairwise comparison of the relative frequencies of BNYVV RNAs between *B. vulgaris* roots infected with BNYVV for 2 w.p.i. (R2W) and 8 w.p.i. (R8W) with the aim of assessing the copy number variation of each genomic RNA according to the infection time. (**E**) Pairwise comparison of the relative frequencies of BNYVV RNAs between *B. vulgaris* roots with and without *P. betae* (R8W and RPb). Asterisks (*) indicate statistical differences in the relative frequencies of the same RNA within the compared sample groups, which were estimated by Wilcoxon rank-sum tests (Table S4; ). The *P*-values less than 0.008 are indicated as *. (**F**) BNYVV SGFs in *B. vulgaris* roots infected with BNYVV for 2 w.p.i. (R2W) and 8 w.p.i. (R8W) and *B. vulgaris* roots infected with BNYVV and *P. betae* (RPb). Numbers indicate the stoichiometric coefficient related to the designated RNA genomic segment in the order of RNA1 : RNA2 : RNA3 : RNA4. BNYVV SGFs were calculated by pooling total RNA extraction data sets. Each RNA stoichiometric coefficient was calculated by dividing the mean of the relative frequency of the RNA by the mean of the relative frequency of the less abundant RNA. SGF values were rounded to the nearest half unit.

We analyzed the viral segment frequencies in *B. vulgaris* roots infected with BNYVV for 2 and 8 weeks (R2W and R8W). Results from the two experimental groups, comprising 12 root samples for R2W and 18 for R8W, indicated that the relative frequencies of each BNYVV RNA changed significantly over time, resulting in two distinct different SGFs: 3 : 2 : 3 : 4 for the R2W SGF and 7 : 2 : 2 : 11 for R8W SGF (Fig. 1A, B, D, and F), where the numbers indicate the stoichiometric coefficient related to the designated RNA genomic segment in the order of RNA1 : RNA2 : RNA3 : RNA4. For both time points, BNYVV RNAs exhibit statistically different relative frequencies (Fig. 1A, B, and D). However, while the differences in relative frequencies are minimal in plants analyzed after 2 weeks post-virus inoculation (p.v.i.), differences become more pronounced 8 weeks p.v.i., with mean relative frequencies of RNA1 and RNA4 being approximately 244% and 456% more abundant, respectively, than those of RNA2, which is the least abundant genome segment in *B. vulgaris*-infected roots. In assessing the impact of *P. betae* on BNYVV accumulation in the host, aviruliferous zoospores were introduced into *B. vulgaris* pots 6 weeks after viral root infection (RPb). Host infection by *P. betae* was verified via PCR analysis of the root nucleic acid extracts. Subsequently, the relative accumulation of BNYVV RNAs in roots was evaluated after 2 weeks in 18 samples. This corresponds to the analysis of plants 8 weeks p.v.i. and infected for 2 weeks by the protozoan. Results revealed a statistically significant alteration only in the accumulation of RNA2, with an approximately 88.89% increase in its relative frequency (Fig. 1E), reaching an SGF of 7 : 4 : 2 : 11 (Fig. 1C and F). However, this difference was deemed marginal from a biological standpoint. In the overall pattern of relative frequencies, RNA1 and RNA4 were significantly more abundant compared to RNA2 and RNA3, which were relatively rarer (Fig. 1C and E).

### Purification protocol validation for *P. betae* zoospores and resting spores

The validation process for the extraction and purification protocol of virulent secondary zoospores and resting spores of *P. betae* from the rhizosphere and roots of *B. vulgaris* underwent thorough quality control procedures. These measures were designed not only to enrich the two distinct life cycle forms of the vector but also to ensure the exclusion of plant-origin contaminants. This was crucial to prevent the quantification of host-derived viral components not acquired by the vector. Given that the *P. betae* syncytial form shares the cytoplasm of the host plant, extending the analysis beyond this compartment was crucial. Therefore, purity control relied on monitoring the presence of *B. vulgaris* nuclear content, which should not be detected in the released *P. betae* zoospores and resting spores.

The enrichment of one form of the protozoan over the other is achieved through different enrichment methods based on the distinct physical properties of zoospores and resting spores. Zoospores are motile and can easily release into the aqueous solution in which the roots of the infected plant have been immersed, while resting spores are more robust and tend to remain trapped as cystosori within the roots or root debris. This difference allows for their effective separation: zoospores are collected from the solution, while resting spores stay anchored to the plant material or are pelleted during the initial low-speed centrifugation steps. In the case of resting spores, the purification method takes advantage of their physical resilience to stress. Resting spores are resistant to freezing, grinding of the tissues, and the mechanical action of sonication. Zoospores, in contrast, are not as resilient; their residues are lost during subsequent filtration and centrifugation steps. This contrast in physical properties is key to selectively enriching each form of the protozoan in our preparations.

**Fig 2:**
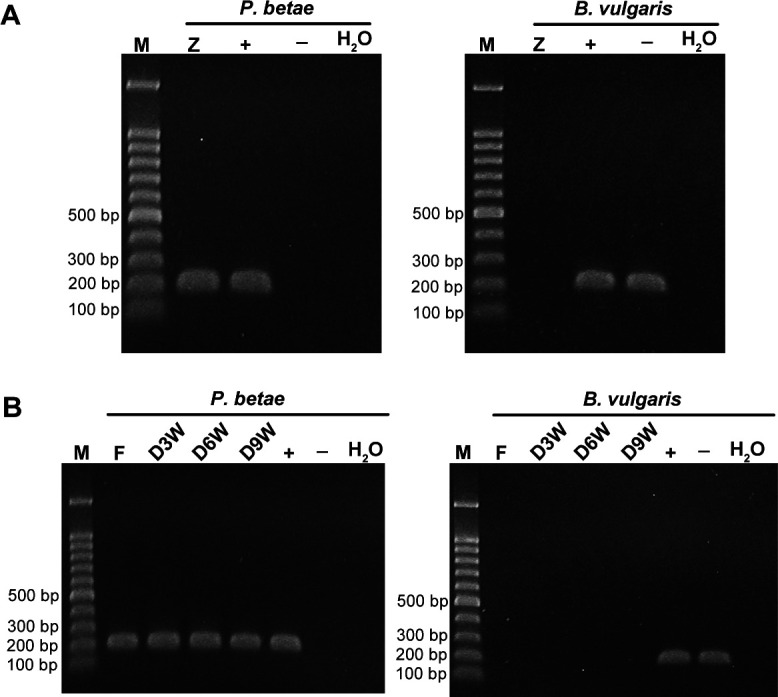
(**A**) Electrophoresis analyses of PCR amplicons specific to *P. betae* ITS1 (left) and *B. vulgaris* GS gene (right) obtained from *P. betae* and *B. vulgaris* in *P. betae* zoospore purification. Z, *P. betae* zoospore purification; positive control (+), *B. vulgaris* roots infected with viruliferous *P. betae*; negative control (−), healthy *B. vulgaris* roots. (**B**) Electrophoresis analysis of PCR-specific fragments (as in panel A) obtained from *P. betae* and *B. vulgaris* in *P. betae* resting spore purifications. F, *P. betae* resting spore purified from fresh *B. vulgaris* roots infected with viruliferous *P. betae*; D3W, *P. betae* resting spore purified from air-dried 3-week *B. vulgaris* roots infected with viruliferous *P. betae*; D6W, *P. betae* resting spore purified from air-dried 6-week *B. vulgaris* roots infected with viruliferous *P. betae*; D9W, *P. betae* resting spore purified from air-dried 9-week *B. vulgaris* roots infected with viruliferous *P. betae*; positive control (+), *B. vulgaris* roots infected with viruliferous *P. betae*; negative control (−), healthy *B. vulgaris* roots.

**Fig 3:**
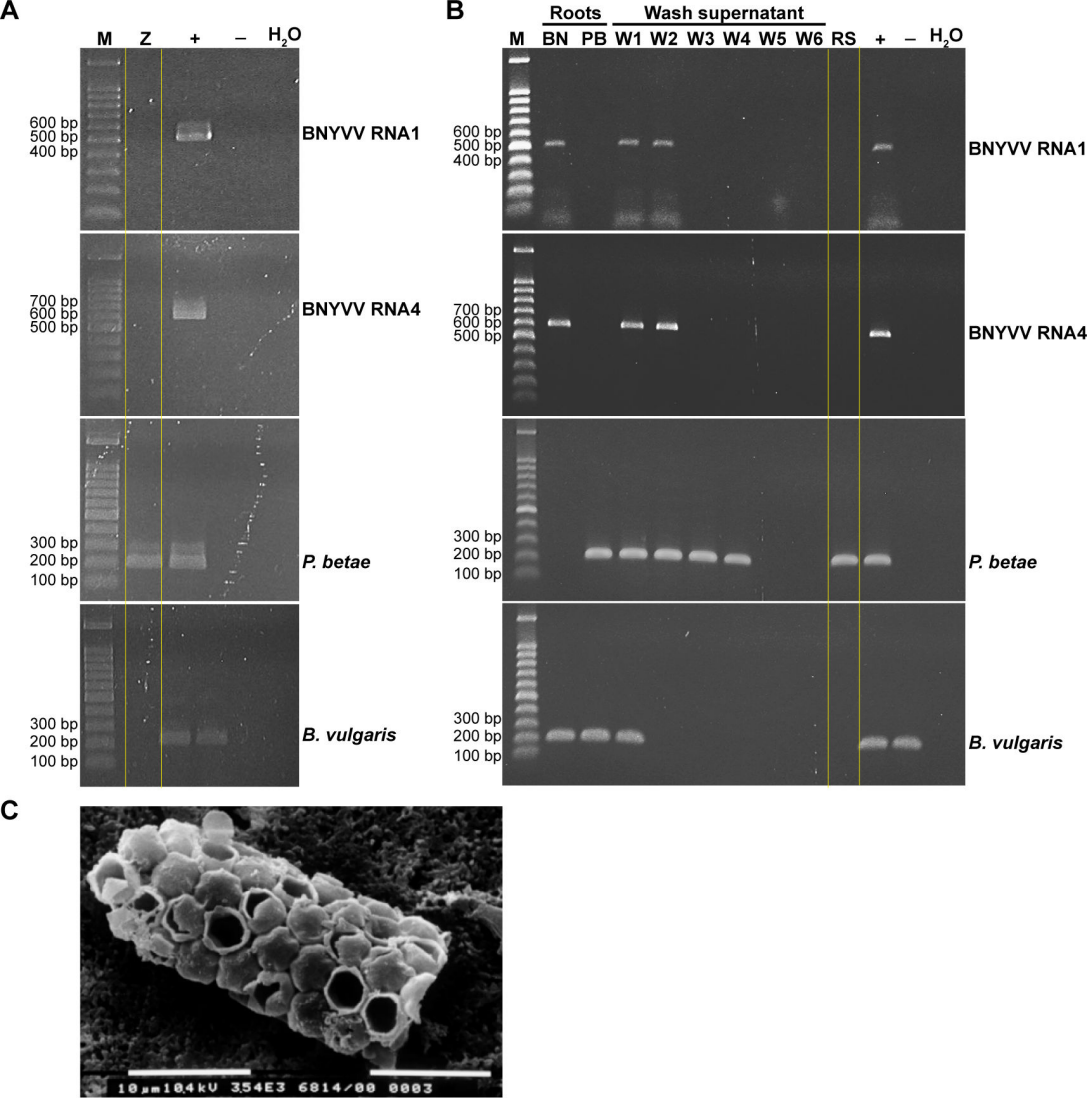
(A and B) Electrophoresis analyses of PCR- or RT-PCR-specific fragments obtained from BNYVV RNA1, RNA4, *P. betae,* and *B. vulgaris* in *P. betae* zoospore (**Z**) and resting spore (RS) purified after the mixture of *B. vulgaris* roots infected by BNYVV (without *P. betae*) (BN) with *B. vulgaris* roots infected with BNYVV-free *P. betae* (PB). W1–W6: supernatant samples after each washing step; positive control (+), *B. vulgaris* roots infected with viruliferous *P. betae*; negative control (−), healthy *B. vulgaris* roots. (**C**) Micrograph obtained by scanning electron microscopy from purified *P. betae* resting spores.

To verify the exclusion of plant-origin contaminants, PCRs targeting the *B. vulgaris* GS gene were performed, resulting in no amplification of the plant nuclear target for the zoospore and resting spore purification, whereas amplicons were detected in infected and non-infected *B. vulgaris* material (Fig. 2). To ensure prevention of any possible accidental contamination of viral genome material on zoospore surfaces during purification, we performed an additional control investigation. We combined root exudates from both BNYVV-infected plants without *P. betae* and plants without the virus but with the vector. After purification of the aviruliferous zoospores from this mixture, we targeted BNYVV RNAs via RT-PCR following nucleic acid extraction. The negative results confirmed the absence of viral nucleic material on the spores’ surfaces, thus validating the effectiveness of our purification method (Fig. 3A; Fig. S1A). Similarly, roots from plants infected solely with the virus and solely with the vector were mixed, and the extraction and purification protocol for resting spores was applied. Once again, the purified nucleic acids showed no detectable viral contamination within the qualitative detection limits of RT-PCR analysis (Fig. 3B; Fig. S1B). Specifically, viral contamination was eliminated starting from the second centrifugation wash, while contamination from *B. vulgaris* DNA material was removed at the beginning of the first wash. This was confirmed by negative results in the RT-PCR reactions for the supernatant obtained from the third and second washes, respectively (Fig. 3B; Fig. S1B). As a precautionary measure, we opted to maintain the five washes outlined in the protocol described in Materials and Methods. Additionally, clusters of purified *P. betae* resting spores (sporosori) were observed under scanning electron microscopy (SEM), confirming the absence of plant tissue residues (Fig. 3C). Interestingly, some of the purified *P. betae* resting spores were found in an open state, suggesting germination and the release of zoospores (Fig. 3C).

### Characterization of BNYVV load and SGF in *P. betae* zoospores and resting spores

**Fig 4:**
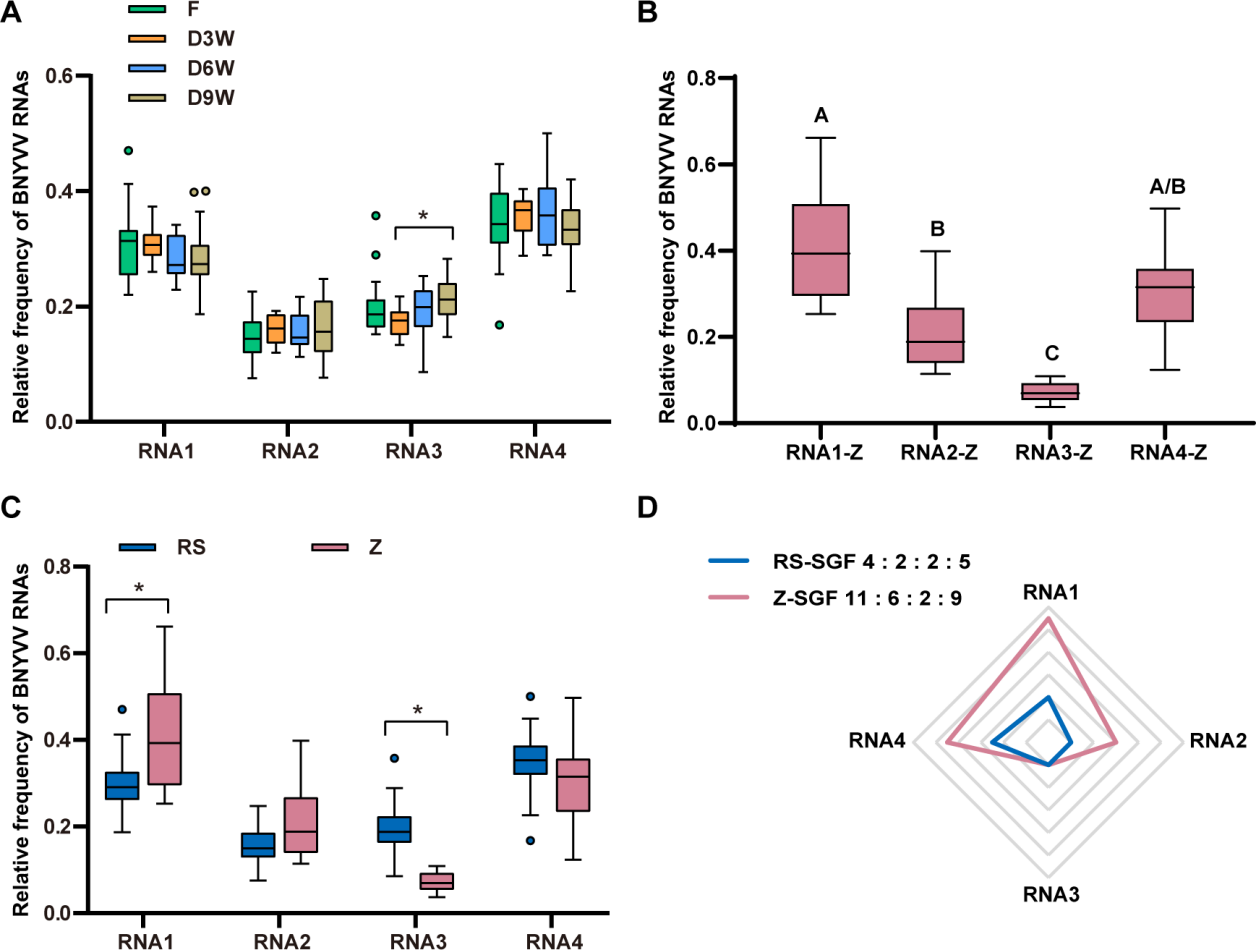
(**A**) Relative frequencies of BNYVV vRNAs in four kinds of *P. betae* resting spores (fresh [F] or dried 3, 6, and 9 weeks after harvest [D3W, D6W, and D9W]). (**B**) BNYVV vRNAs relative accumulation in *P. betae* zoospores. (**C**) Pairwise comparison of the relative frequencies of BNYVV RNAs between *P. betae* resting spores and zoospores. RS, *P. betae* resting spore purification (the combination of the four resting spore data sets described above); Z, *P. betae* zoospore purification. (**D**) BNYVV SGFs in *P. betae* zoospores and resting spores. The BNYVV SGF of resting spores was calculated from the combination of the four resting spore data sets.

The relative frequencies of BNYVV RNAs were assessed in purified *P. betae* resting spores collected from both fresh *B. vulgaris* roots (F) and those air-dried for 3, 6, and 9 weeks (D3W, D6W, and D9W, respectively), with each group comprising 20 samples. Statistical analysis using Wilcoxon rank-sum tests revealed no significant differences in the relative accumulation of each BNYVV RNA across different drying times (Fig. 4A; Table S4). Consequently, data from all four datasets were combined to compute a BNYVV RS-SGF (resting spores Setpoint Genome Formula of 4 : 2 : 2 : 5) (Fig. 4C and D). The data used to calculate the BNYVV SGF of zoospores (Z) were obtained from the analysis of 12 samples of purified zoospores. In this case as well, RNA1 and RNA4 were identified as the two most abundant genomic segments, displaying a pronounced difference in their relative frequency as compared to RNA3 (Fig. 4B and C). This resulted in a Z-SGF of 11 : 6 : 2 : 9 (Fig. 4D).

**Fig 5:**
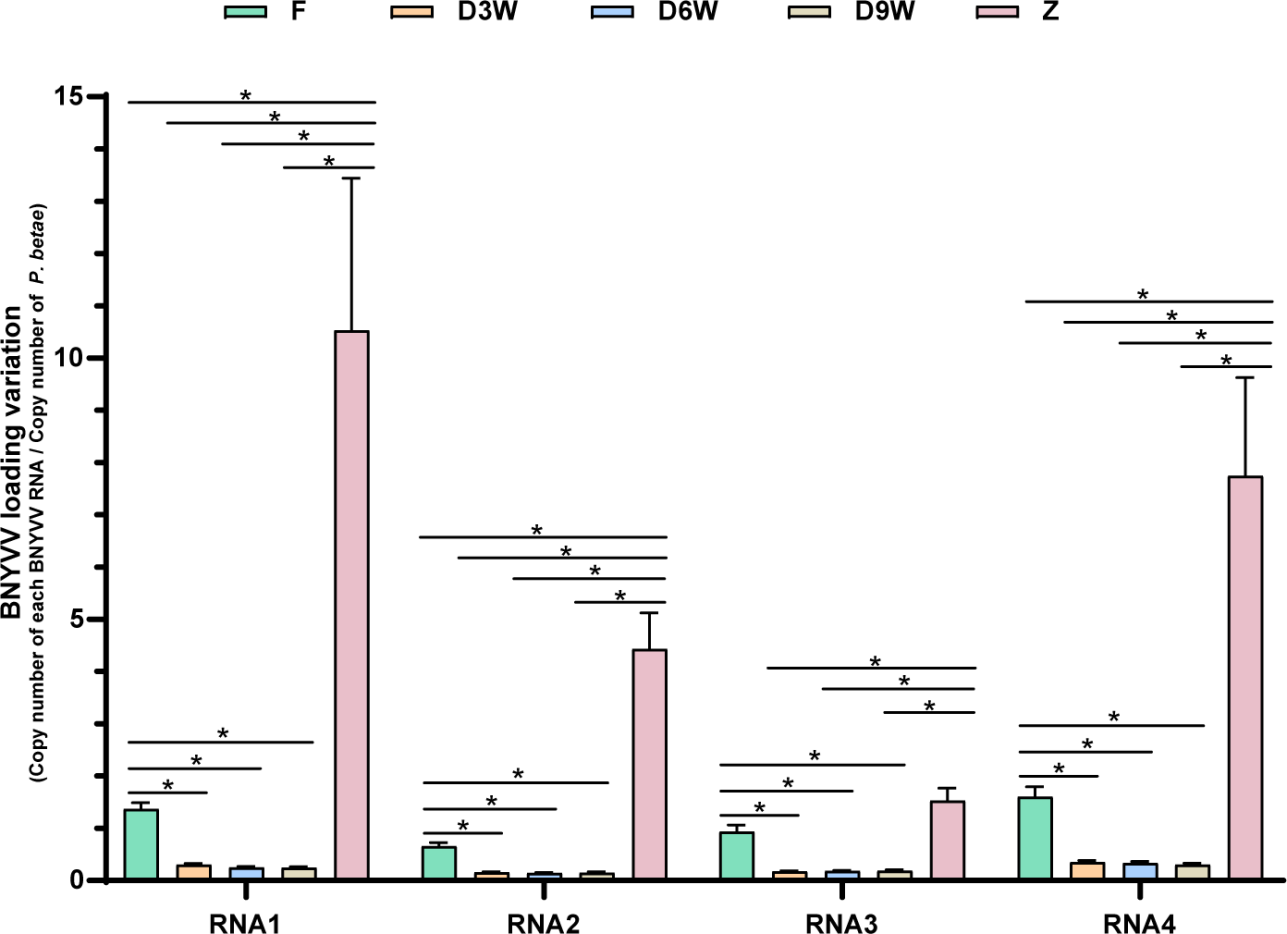
BNYVV loads in four kinds of *P. betae* resting spores and zoospores. F, *P. betae* resting spore purified from fresh *B. vulgaris* roots infected with viruliferous *P. betae*; D3W, *P. betae* resting spore purified from air-dried 3-week *B. vulgaris* roots infected with viruliferous *P. betae*; D6W, *P. betae* resting spore purified from air-dried 6-week *B. vulgaris* roots infected with viruliferous *P. betae*; D9W, *P. betae* resting spore purified from air-dried 9-week *B. vulgaris* roots infected with viruliferous *P. betae*; Z, *P. betae* zoospore purification. Asterisks (*) indicate statistical differences in loads of the same RNA within the compared sample groups, which were estimated by Wilcoxon rank-sum tests (Table S5). The *P*-values less than 0.008 are indicated as *.

By infecting *B. vulgaris* plants with viruliferous *P. betae* and then extracting both resting spores and secondary zoospores from the same group of infected plants, it is likely that both types of spores carry the virus or have a similar proportion of viruliferous spores. This is because both zoosporangia and cystosori originate from the same viruliferous plasmodia. This assumption is crucial for accurately interpreting the subsequent analysis of viral titer dynamics in the *P. betae* life cycle. We chose to utilize the DNA component instead of RNAs from *P. betae*. Indeed, we needed to correlate the virus quantity with the number of resting spore or zoospore cells, considering the likely differences in transcriptomes, both in terms of quantity and quality, between the two different stages of the protozoan life cycle. Therefore, to evaluate the viral titer in viruliferous resting spores and zoospores, we opted to standardize the BNYVV RNA CNs obtained from RT-ddPCR analysis of the nucleic acid extracts with the internal transcribed spacer 1 (ITS1) CNs of *P. betae* acquired from ddPCR analysis. This rationale draws parallels with studies conducted on other plasmodiophorids (42). The kinetic analysis of viral titers in nucleic acid extracts from resting spores, subjected to varying durations of natural drying treatment, revealed a notable trend: a progressive decrease in BNYVV RNA concentrations over time (Fig. 5), while their relative frequencies remained unaffected (Fig. 4A). Notably, during the initial observed time interval following 3 weeks of drying, the viral titer remarkably declined by approximately 78%, as depicted in Fig. 5. Furthermore, when looking at RNA3 and RNA1, which correspond, respectively, to the less and the most abundant BNYVV genomic RNA segments within zoospores, it becomes clear that the RNA levels in this protozoan form increase significantly by 62.77% and 669.34% compared to the levels in resting spores purified and enriched from fresh tissues (Fig. 5).

## DISCUSSION

This is the first report for the investigation of the relative RNA frequencies of BNYVV in the roots of its natural host, *B. vulgaris,* and more importantly in two living forms of its vector, *P. betae*. Our analyses unveiled differences compared to those observed in the roots of *Beta macrocarpa* and *Spinacea oleracea*, where the virus reached different SGFs (18). Notably, in *B. vulgaris* roots, BNYVV RNA frequencies can fluctuate at different stages of infection and are unaffected by the presence of aviruliferous *P. betae,* at least when zoospores are introduced into the rhizosphere of plants previously infected by the virus for 6 weeks.

Experiments conducted on *Chenopodium quinoa* protoplasts have shown that BNYVV reaches its peak viral titer 48 hours p.v.i., with preservation observed until 72 hours p.v.i., followed by a reduction of the titer likely due to protoplast lysis and cell degeneration in the culture media within subsequent hours (43). Therefore, it is expected that a similar pattern may occur in *B. vulgaris* root cells, starting with the expansion of primary infection sites, followed by the systemic spread of the infection. Initially, most infected cells exhibit early-stage viral infection. As the infection progresses, the proportion of cells showing early-stage viral infection diminishes, while the proportion of cells with mature-stage viral infection rises. An example illustrating how the genomic formula of a multipartite RNA virus can change in infected host tissues as viral infection progresses is provided by RSV in rice (15). During a 20-day study period, RSV genomic RNAs exhibited fluctuating relative frequencies, influenced by the fact that each genomic component reached its peak titer at different times (15). These findings are corroborated by a recent study on FBNSV, which suggested that achieving a specific SGF is not solely determined by the individual replication rate of each genomic element in a particular infectious context but also by group-level processes influenced by the collective behavior of the different segments (44). Therefore, this concept can be extended to the idea that during infection, the relative abundance of one genomic element may influence that of another in an orchestration leading to the convergence toward a specific SGF. In the tripartite system of virus, *B. vulgaris*, and *P. betae*, it has been demonstrated that starting from 2 weeks after the inoculation of zoospores, the protozoan reaches the maximum level of primary root colonization with plasmodium production (45). During this period, it is described as an upregulation of plant genes such as those expressing PR proteins, proteins involved in the synthesis of phenolic compounds, and other genes related to defense pathways (45). These transcriptomic changes, under our experimental conditions, seem not to interfere with the regulation of the relative frequencies of BNYVV. Hence, BNYVV conserves its SGF in the roots of *B. vulgaris*, regardless of the presence of the protozoa.

Our findings on BNYVV RNA titers in secondary zoospores and resting spores align with earlier studies (46, 47), which demonstrated detectable viral particles in the plasmodial form, zoosporangia, and zoospores, albeit in lower concentrations compared to adjacent infected *B. vulgaris* root cells, but not in mature resting spores, probably due to the low virus quantity in these stages. Our RT-ddPCR analyses reveal a significant decrease in the viral RNA titer in resting spores compared to secondary zoospores. Additionally, as resting spores undergo the drying process, mimicking summer environmental conditions, the titer continues to decrease until it stabilizes at a lower level. Despite such reduction, it has been demonstrated that the stabilized titer in dried resting spores remains sufficient to ensure BNYVV infectivity in the primary zoospores that will germinate, albeit limited by the estimated requirement of at least 150 resting spore clusters for effective transmission to a new *B. vulgaris* plant (48). Consequently, it has been suggested that the true productive dissemination of BNYVV in a sugar beet field occurs through the propagation of viruliferous secondary zoospores (47, 49).

In addition to differing viral titers in resting spores and zoospores, our experiments indicate that BNYVV reaches different SGFs in these protozoan life cycle stages, which also differ from those detected in the roots of *B. vulgaris*. While the different SGFs of BNYVV in various host plants have been explained by processes related to the replication of its genomic components (18), understanding the reasons behind the differing frequencies observed in the two forms of *P. betae* remains challenging, as it is still controversial whether the virus possesses the capacity to replicate in its vector. The difference between RNA1 and RNA4, the most abundant genomic components, and RNA3, the least abundant, becomes more pronounced in zoospores. This likely reflects the virus’s need to focus on replication and silencing suppression during the early stages of infection after transmission to a new host or in a new infection site within the same host.

Assuming that all viral RNA segments have a similar efficiency in being translated, the higher relative abundance of RNA1 and RNA4 suggests that the virus prioritizes these functions at the onset of infection. High levels of RNA1 ensure efficient replication, while RNA4 helps suppress the plant’s antiviral defense mechanisms. This enables the virus to simultaneously replicate and evade host defenses. In contrast, RNA3, which is much less abundant, plays a different role. Its product, p25, can trigger the plant’s hypersensitive response, leading to localized cell death to limit viral spread (50, 51). Therefore, RNA3 expression must be tightly regulated. Overexpression of RNA3 at an early stage could prematurely activate plant defenses, causing cell death and limiting the virus’s ability to spread. Once the virus has established itself in the host, RNA3 expression can increase, facilitating the virus’s systemic movement throughout the plant without causing premature host cell death.

Given the dormant nature of resting spores, it is plausible that the virus’s SGF in this stage depends on the different half-lives of its genomic components. Conversely, the higher viral titer and distinct SGF observed in zoospores may suggest viral activity in this alternative plasmodiophorid form. However, due to the lack of data on the replicative kinetics of the virus in zoospores, given their short half-life, robust support for this speculation is challenging. Another plausible explanation is that the SGF of BNYVV in zoospores could be influenced by selective processes involving its genomic components during viral acquisition by *P. betae* in the plasmodium formation stage, independent of viral replication. The mechanism of viral acquisition by *P. betae* is still far from being fully understood. It has been determined that the BNYVV p31 protein enhances the viral transmission from one plant to another via *P. betae* (37). Additionally, the virus’s encapsidation has historically been considered a necessary condition for transmission, as p75 mutants that are defective in encapsidation are not transmitted or poorly transmitted by the vector (52). The same occurs for mutants that retain encapsidation ability but possess deletions or alterations in the KTER motif of the minor structural protein, suggesting that these four amino acids are directly involved in virus-protozoan membrane contacts during transmission (53, 54) coordinating the association of two putative transmembrane helices of the protein (55). In these experiments, however, viral transmission was assessed through the analysis of the viral population in bait plants, making it unclear how p31, p75, or the encapsidation process contributes to the viral acquisition by the vector, as this stage was not separated from others that together constitute the transmission process. The observation of vesicles in young immature plasmodia or zoospores containing viral particles and protein factors (41, 46) may suggest that the virus may be acquired through pinocytotic processes, wherein portions of the host cytoplasm containing ribonucleoprotein (RNP) complexes are taken up by the vector. Although the model we propose currently lacks direct scientific evidence, it aligns with studies on other RNA soil-borne viruses transmitted by *Polymyxa* species, which suggest that these viruses may be internalized and transmitted by the protozoan not as virion particles but as RNP complexes (56). These RNP complexes indeed constitute collective infectious units (31, 57) that allow the multipartite virus to maintain its genomic integrity within the vector, ensuring horizontal transmission. The genomic component of the complexes at the time of acquisition may already exhibit the SGF observed in zoospores or develop it later within the vector’s plasmodial form, where the presence of host-derived proviral replicative factors internalized by *P. betae* could support some viral replication cycles.

We emphasize once more that these speculations are not yet supported by evidence and extend beyond the scope of our published work. However, the analyses of relative frequencies and genomic RNA loading of BNYVV in *P. betae* surely raise questions regarding how the virus is acquired by the vector and whether it undergoes replication within it. For the first time, our study introduces a protocol for purifying and enriching viruliferous zoospores and resting spores, along with nucleic content analysis via digital PCR. This advancement now enables reverse genetics experiments with mutants, specifically focusing on the initial stages of viral transmission, thereby decoupling the acquisition of the virus by the protozoan from its injection into a new host cell.

## MATERIALS AND METHODS

### Host plant and viral inoculation

Rhizomania-susceptible *Beta vulgaris* cultivar (Portland) was used as the host plant in all experiments. The plants were grown with a 16/8-h day/night photoperiod, a temperature of 22°C/18°C day/night, and a constant 70% humidity. In experiments aimed at calculating BNYVV SGF in *B. vulgaris* roots, in the absence or presence of aviruliferous *P. betae*, plant seeds were sown in pots and grown in sterile sand. Seven-day-old seedlings were collected, and their roots were washed with sterile distilled water. Roots were then immersed in the inoculum consisting of infected sap from *C. quinoa* leaves previously finely ground in KH_2_PO_4_ buffer (10 mM) containing carborundum powder (30 mg/mL). Viral infection was facilitated by vortexing for 1 minute, followed by an ice bath for 1 minute. The seedlings were then transplanted back into pots filled with sterile sand.

The *P. betae* isolate used in these experiments originated from the collection of aviruliferous zoospores extracted from an infected *B. vulgaris* plant recovered in the province of Bologna, Italy. This isolate, which originated in the 1990s, has been maintained in the laboratory since then. The soil infested with *P. betae* was obtained by releasing aviruliferous zoospores from infected *B. vulgaris* roots. Specifically, infected roots were collected from a group of 20 2-month-old plants and submerged in bovine serum albumin (BSA, Sigma Aldrich, Product No. 05470) 0.5% solution for 4 hours. The virus-free zoospore solution was then introduced into pots housing 7-week-old *B. vulgaris* plants, which had been infected mechanically with BNYVV 6 weeks earlier. Sampling was conducted at various intervals: roots infected solely by BNYVV were harvested at 2 and 8 weeks post-virus inoculation, while roots infected by BNYVV and grown in soil infested with avirulent *P. betae* were collected at 8 weeks p.v.i.

### *P. betae* spore purifications

Both viruliferous zoospores and resting spores were released from *B. vulgaris* roots of plants 2 months after being infected with the viruliferous *P. betae*. Specifically, *B. vulgaris* seeds were germinated, and seedlings were grown in soil containing dried roots harboring viruliferous resting spores. Each zoospore sample was released by soaking the roots of 20 plant bunches in 50 mL of a 0.5% BSA solution for 4 hours. Subsequently, the zoospore solution was purified through three successive centrifugations: first at 9,384 × *g* for 5 minutes, then at 2,599 × *g* for 30 minutes, and finally at 70,000 × *g* for 60 minutes to obtain a pellet. Bulked samples of roots were divided into four equal samples: one was treated immediately and the other three were left to air dry on the bench at room temperature. One sample was left for 3 weeks, another for 6 weeks, and the last one for 9 weeks. Resting spores were extracted from these four root bunch samples as follows: 200 mg of roots was ground using liquid nitrogen, and the homogenate was suspended in 2 mL of nuclease-free water and sonicated at 12 KHz to enhance the release of resting spores from the plant tissues. After filtration with 20 layers of non-woven fabric (30 g/m^2^), the resting spores were washed five times with nuclease-free water and pelleted through centrifugation cycles at 9,184 × *g* for 5 minutes. Twenty resting spore purifications were obtained from fresh and air-dried roots for 3, 6, and 9 weeks (designated as F, D3W, D6W, and D9W, respectively).

### Nucleic acid extraction and analyses

Two hundred milligrams of *B. vulgaris* roots, along with the purified pellets of zoospores and resting spores, and 500 µL of the supernatant from each washing step performed during the spore purification protocol validation underwent total nucleic acid extraction using a modified cetyltrimethylammonium bromide (CTAB) extraction method (58). Briefly, the samples were homogenized in 1 mL of CTAB buffer (2.0% CTAB, 100 mM Tris-HCl pH 8.0, 20 mM EDTA, 1.4 M NaCl, 1.0% Na sulfite, and 2.0% PVP-40) and incubated at 65°C for 30 minutes. Subsequently, a phase separation purification was carried out by adding 400 µL of chloroform:isoamyl alcohol (24:1), followed by precipitation with one volume of cold isopropanol (50%). The resulting pellets were then suspended in 400 µL of TE buffer (10 mM Tris-HCl pH 8 and 1 mM EDTA) and subjected to a second round of phase separation purification using an equal volume of chloroform:isoamyl alcohol (24:1). Precipitation was achieved by adding two volumes of 100% ethanol and 1/10 volume of 3 M sodium acetate (pH 5.2) to the aqueous phase. After washing with 70% ethanol and air drying, the nucleic acid pellets were resuspended in 40 µL of nuclease-free water, and RNA content was quantified using a Qubit 3.0 fluorometer with RNA Broad-Range Assay Kit (Life Technologies). RNA concentrations in the samples were adjusted to 5 ng/µL for *B. vulgaris* root and 200 pg/µL for *P. betae* zoospore and resting spore nucleic acid purifications. A volume of 1 µL of each sample was then used as a template for qualitative analysis via PCR or RT-PCR, as well as quantitative analysis via ddPCR or two-step RT-ddPCR of *B. vulgaris* and *P. betae* DNA, and BNYVV genomic RNA, using the primers listed in Tables 1 and 2. All two-step RT-ddPCR reactions for quantifying BNYVV RNAs followed the protocol outlined by Dall’Ara et al. (18). The relative frequency of each BNYVV RNA segment was calculated following the method described by Dall'Ara et al. (18). Specifically, the copy number of each BNYVV genomic RNA was divided by the sum of all quantified genomic RNA copy numbers within the same sample. This gives the relative frequency of each segment. Subsequently, BNYVV SGFs of *B. vulgaris* roots and *P. betae* zoospores and resting spores were calculated by dividing the mean relative frequency of each RNA segment by the mean relative frequency of the least abundant segment. The resulting SGF values were then rounded to the nearest half unit. *P. betae* was quantified using an EvaGreen-based ddPCR assay (BioRad) with primers targeting the internal transcribed spacer 1 (ITS 1) (GenBank: HE860064.1) (Table 2). This involved using 1 µL of template nucleic acid extraction and the 2× ddPCR Supermix for EvaGreen (Bio-Rad) with thermal cycling conditions in accordance with the manufacturer’s instructions. The titer of each BNYVV RNA in *P. betae* spores was then calculated through the ratio of the RNA copy number to the ITS1 copy number. Qualitative endpoint PCRs were executed using the GoTaq G2 DNA Polymerase kit from Promega following the manufacturer’s instructions. Specifically, 1 µL of nucleic acid extract was used for the detection of *B. vulgaris* or *P. betae*. For *B. vulgaris* DNA analysis, we utilized primers designed for the glutamine synthetase gene (GS) (GenBank: EU370974.1) (59) (Table 1), while for *P. betae* DNA analysis, we utilized the previously mentioned primers targeting ITS1. Regarding BNYVV RNAs, PCRs were carried out using the reverse transcription product of 1 µL of nucleic acid extract, following the protocol outlined by Dall’Ara et al. (18) with primers listed in Table 2.

**TABLE 1:** Sequences of primers used for RT-PCR and PCR

Target	Primer (sequence 5′-3′)	Amplicon length (bp)
BNYVV RNA1	6150F (GCATTTTTGTGAATACCAGG)	502
	6651R (GTACCACATAATCAAGAACC)	
BNYVV RNA2	19F (CCATTGAATAGAATTTCACC)	1,070
	1088R (CCCCATAGTAATTTTAACTC)	
BNYVV RNA3	409F (AAGTTGTTGTGTTTTCTGAT)	860
	1268R (CGTGAAATCACGTGTAGTTT)	
BNYVV RNA4	699F (TTGATGTTCTGTCTGATGAG)	603
	1301R (CACATAAACCTTACCATAGC)	
*P. betae* (internal transcribed spacer 1)	Pb1 (GGAATTTGAACAAGTGACTTGG)	204
	Psp2rev (AGGGCTCTCGAAAGCGCAA)	
*B. vulgaris* (glutamine synthetase gene)	GSBvFor (AGGGTGATTGGAATGGTGCT)	201
	GSBvRev (ACTTCTCGATGGCAGCCTTT)	

**TABLE 2:** Sequences of primers and probes used for RT-ddPCR and ddPCR

Target	Primer (sequence 5′-3′)	Taq-Man probe-fluorophore (sequence 5′-3′)
BNYVV RNA1	3946F (TGGTTTCACAAGGAGATGTCGTT)	3944 FAM-TTTTGGACATAGCACGTGTGGAAAACGATA
	4024R (TCTGCACAATCAAAGGCATCA)	
BNYVV RNA2	662F (TGGACCCGGGATAAATTTGA)	653 HEX-ACCGGTTCAAATTACCATGGACACCTGTT
	734R (CGGGTGGACTGGTTCTACCTT)	
BNYVV RNA3	54F (ATATGTGAGGACGCTAGCCTGTT)	84 FAM- CTGACCGACCAAATCCAAGCGAGCTTAAT
	172R (TGAAACGATGGAGTCACTATGCTT)	
BNYVV RNA4	415F (TCCTCCTTTGATACGTCATGAAGA)	445 HEX-TGATTGTACTGCTAGGATGGTGCA
	490R (CAATGGGCCAATCTCAATCC)	
*P. betae*	Pb1 (GGAATTTGAACAAGTGACTTGG)	
	Psp2rev (AGGGCTCTCGAAAGCGCAA)	

### Scanning electron microscopy

SEM was used to observe purified resting spores following Sayama et al.’s protocol (60) with slight modifications. Briefly, resting spores were fixed in 0.1 M phosphate buffer (pH 7.2) containing 5% glutaraldehyde for 48 hours. After fixation, samples were rinsed thrice with the same buffer, dehydrated in an ethanol series (30%−100%), and then collected on a nucleopore filter membrane with 5.0 µm porosity. The specimen was subsequently subjected to critical point drying, sputter coating, and observation using a Philips SEM 515 at 15 kV, along with a Pentax K-5 camera for micrograph acquisition.

### Statistical analysis

All statistical tests were conducted using R 4.3.1 software. The normality of the data set was assessed with the Shapiro-Wilk test, indicating that several variables deviated from a normal distribution (Table S1). Consequently, non-parametric tests were employed for analysis. We utilized Friedman’s test to assess significant variation among the dependent variables, which are the BNYVV RNA relative frequencies used to compute each BNYVV SGFs (Table S2). Subsequently, pairwise comparisons of these RNA relative frequencies, including RNA1–RNA2; RNA1–RNA3; RNA1–RNA4; RNA2–RNA3; RNA2–RNA4; and RNA3–RNA4, were conducted using the Wilcoxon signed-rank test with Bonferroni correction for multiple pairwise comparisons, adjusting the significant *P*-value to 0.008 (Table S3). Conversely, Wilcoxon rank-sum tests were employed to compare the relative frequencies and loads of the same RNA across different experimental conditions. In this context, we opted to adjust the significant *P*-value to 0.008 to mitigate the likelihood of incorrectly rejecting a true null hypothesis, thereby upholding a more conservative statistical approach (Tables S4 and S5).

## Data Availability

All relevant data supporting our findings are included within the article and its supplemental material.

## SUPPLEMENTAL MATERIAL

Supplemental material jvi.01410-24-s0001.pdf Statistical analysis and electrophoresis analysis.

ASM does not own the copyrights to Supplemental Material that may be linked to, or accessed through, an article. The authors have granted ASM a non-exclusive, world-wide license to publish the Supplemental Material files. Please contact the corresponding author directly for reuse.
