# Supplementary material for: Relative frequency dynamics and loading of beet necrotic yellow vein virus genomic RNAs during the acquisition by its vector Polymyxa betae
Source: J Virol. 2024 Dec 16;99(1):e01410-24. doi: 10.1128/jvi.01410-24 (PMC11784302; doi:10.1128/jvi.01410-24)
Supplement: Supplemental material — Statistical analysis and electrophoresis analysis. [file jvi.01410-24-s0001.pdf]

Supplementary Table

Table S1. Output of the Shapiro-Wilk test for normality, conducted to assess the distribution of relative frequencies of RNA in *B. vulgaris* roots infected with BNYVV for 2- and 8 weeks (R2W and R8W), *B. vulgaris* roots infested with BNYVV and the vector (RPb) and *P. betae* zoospores (Z). The *p*-values from the test results are displayed, with values less than 0.05 highlighted in red. A *p*-value below 0.05 indicates a normality deviation, implying non-parametric data for that specific category.

	**RNA1**	**RNA2**	**RNA3**	**RNA4**
***B. vulgaris*-R2W**	0.0150	0.7637	0.3284	0.7524
***B. vulgaris*-R8W**	0.0198	0.0309	0.0578	0.0036
***B. vulgaris*-RPb**	0.0494	0.4883	0.5905	0.8762
***P. betae*-Z**	0.4916	0.1725	0.6677	0.9844

Table S2. Output of the Friedman test used to evaluate the presence of a statistical difference among the relative frequencies of genomic RNAs employed to calculate BNYVV SGF in the total RNA extracted from *B. vulgaris* roots infected with BNYVV for 2- and 8 weeks (R2W and R8W), *B. vulgaris* roots infested with BNYVV and the vector *P. betae* (RPb), as well as from *P. betae* zoospores and resting spores purified from fresh *B. vulgaris* roots, air-dried 3-, 6- and 9 weeks *B. vulgaris* roots carrying with viruliferous *P. betae* (F, D3W, D6W and D9W). The output includes sample size (N), chi-squared value (χ^2), degrees of freedom (df), and *p*-value. The *p*-values below 0.05 indicate the rejection of the null hypothesis are marked in red, highlighting the presence of at least one statistically significant difference among the relative frequencies of genomic RNAs.

	**R2W**	**R8W**	**RPb**	**Z**	**F**	**D3W**	**D6W**	**D9W**
**N**	12	18	18	12	20	20	20	20
**χ^2**	79.6	192.8	211.47	102.96	177.47	209.76	193.27	165.36
**df**	3	3	3	3	3	3	3	3
**p-value**	< 2.2e-16	< 2.2e-16	< 2.2e-16	< 2.2e-16	< 2.2e-16	< 2.2e-16	< 2.2e-16	< 2.2e-16

Table S3. Output of the Wilcoxon signed-rank test used to assess statistical differences in the relative frequencies of BNYVV genomic RNAs, which were used to calculate the viral SGF in roots of *B. vulgaris* infected with BNYVV for 2- and 8 weeks (R2W and R8W), *B. vulgaris* roots infested with BNYVV and the vector (RPb) and *P. betae* zoospores (Z). The two tailed *p*-values from the test results are displayed and values less than 0.008 are marked in red and are considered statistically significant. This implies a rejection of the null hypothesis and indicates significant differences in the variables under examination. The analysis was used to determine whether the relative frequency groups of BNYVV genomic RNAs are homogeneous or heterogeneous, as depicted by the letters in the box plot in Fig. 1 and Fig. 4.

	**RNA2-RNA1**	**RNA3-RNA1**	**RNA4-RNA1**	**RNA3-RNA2**	**RNA4-RNA2**	**RNA4-RNA3**
***B. vulgaris*-R2W**	0.0034	0.9697	0.0093	0.0522	0.0005	0.0049
***B. vulgaris*-R8W**	< 0.0001	< 0.0001	< 0.0001	0.0665	< 0.0001	< 0.0001
***B. vulgaris*-RPb**	< 0.0001	< 0.0001	< 0.0001	< 0.0001	< 0.0001	< 0.0001

P. betae-Z	0.0068	0.0005	0.1230	0.0005	0.0342	0.0005

Table S4. Outputs from the Wilcoxon rank-sum tests used to compare the relative frequencies of BNYVV RNAs across various study groups. The test was conducted in pairs for comparisons between the frequencies of RNA1s, RNA2s, RNA3s, and RNA4s (RNA1-RNA1, RNA2-RNA2, RNA3-RNA3 and RNA4-RNA4) to assess the statistical differences between the two groups that were compared each time. The results include the W-value and *p*-value for each comparison. Significant differences, indicated in red, are *p*-values that fall below 0.008. R2W and R8W: *B. vulgaris* roots infected with BNYVV for 2- and 8 weeks; RPb: *B. vulgaris* roots infested with BNYVV and the vector; F: resting spores purified from fresh *B. vulgaris* roots carrying with viruliferous *P. betae*; D3W, D6W and D9W: resting spores purified from air-dried 3-, 6- and 9 weeks *B. vulgaris* roots carrying with viruliferous *P. betae*; RS: the combination of the four resting spore purifications datasets; Z: zoospore purifications.

R2W vs R8W

	RNA1-RNA1	RNA2-RNA2	RNA3-RNA3	RNA4-RNA4
**W**	56	208	201	15
***p*-value**	0.02760272	1.549254e-06	1.565441e-05	1.565441e-05

R8W vs RPb

	RNA1-RNA1	RNA2-RNA2	RNA3-RNA3	RNA4-RNA4
**W**	222	17	210	190
***p*-value**	0.05914718	2.671035e-07	0.1341395	0.3888324

F vs D3W

	RNA1-RNA1	RNA2-RNA2	RNA3-RNA3	RNA4-RNA4
**W**	208	144	268	165
***p*-value**	0.841	0.1344	0.0675	0.3547

F vs D6W

	RNA1-RNA1	RNA2-RNA2	RNA3-RNA3	RNA4-RNA4
**W**	246	157	183	169
***p*-value**	0.2211	0.2534	0.6588	0.4135

F vs D9W

	RNA1-RNA1	RNA2-RNA2	RNA3-RNA3	RNA4-RNA4
**W**	248	155	150	214
***p*-value**	0.2012	0.2315	0.1826	0.718

D3W vs D6W

	RNA1-RNA1	RNA2-RNA2	RNA3-RNA3	RNA4-RNA4
**W**	282	209	120	196
***p*-value**	0.02633	0.8201	0.03041	0.9254

D3W vs D9W

	RNA1-RNA1	RNA2-RNA2	RNA3-RNA3	RNA4-RNA4

**W**	286	182	84	257
***p*-value**	0.01954	0.6395	0.001291	0.1274

D6W vs D9W

	**RNA1-RNA1**	**RNA2-RNA2**	**RNA3-RNA3**	**RNA4-RNA4**
**W**	191	178	162	240
***p*-value**	0.8201	0.5648	0.3141	0.2888

RS vs Z

	**RNA1-RNA1**	**RNA2-RNA2**	**RNA3-RNA3**	**RNA4-RNA4**
**W**	204	308	957	642
***p*-value**	0.001403	0.04678	3.308e-08	0.06116

Table S5. Outputs from the Wilcoxon rank-sum tests used to compare the loads of BNYVV RNAs across various study groups. The test was conducted in pairs for comparisons between the frequencies of RNA1s, RNA2s, RNA3s, and RNA4s (RNA1-RNA1, RNA2-RNA2, RNA3-RNA3 and RNA4-RNA4) to assess the statistical differences between the two groups that were compared each time. The results include the W-value and *p*-value for each comparison. Significant differences, indicated in red, are *p*-values that fall below 0.008. F, D3W, D6W, D9W and Z are described as above.

F vs D3W

	**RNA1-RNA1**	**RNA2-RNA2**	**RNA3-RNA3**	**RNA4-RNA4**
**W**	387	382	393	380
***p*-value**	5.411816e-09	2.31707e-08	6.529000e-10	3.937713e-08

F vs D6W

	**RNA1-RNA1**	**RNA2-RNA2**	**RNA3-RNA3**	**RNA4-RNA4**
**W**	392	378	390	380
***p*-value**	9.720956e-10	6.531902e-08	2.016736e-09	3.937713e-08

F vs D9W

	**RNA1-RNA1**	**RNA2-RNA2**	**RNA3-RNA3**	**RNA4-RNA4**
**W**	389	379	388	385
***p*-value**	2.829233e-09	5.083915e-08	3.946418e-09	9.924080e-09

F vs Z

	**RNA1-RNA1**	**RNA2-RNA2**	**RNA3-RNA3**	**RNA4-RNA4**
**W**	1	0	66	19
***p*-value**	1.771535e-08	8.857677e-09	0.035882	1.782165e-05

D3W vs D6W

	**RNA1-RNA1**	**RNA2-RNA2**	**RNA3-RNA3**	**RNA4-RNA4**
**W**	253	225	188	220
***p*-value**	0.157160	0.511661	0.758351	0.601662

D3W vs D9W

	RNA1-RNA1	RNA2-RNA2	RNA3-RNA3	RNA4-RNA4
**W**	262	243	175	245
***p*-value**	0.096500	0.253380	0.511661	0.231499

D3W vs Z

	RNA1-RNA1	RNA2-RNA2	RNA3-RNA3	RNA4-RNA4
**W**	0	0	0	0
***p*-value**	8.857677e-09	8.857677e-09	8.857677e-09	8.857677e-09

D6W vs D9W

	RNA1-RNA1	RNA2-RNA2	RNA3-RNA3	RNA4-RNA4
**W**	203	214	188	266
***p*-value**	0.946695	0.717950	0.758351	0.494523

D6W vs Z

	RNA1-RNA1	RNA2-RNA2	RNA3-RNA3	RNA4-RNA4
**W**	0	0	0	0
***p*-value**	8.857677e-09	8.857677e-09	8.857677e-09	8.857677e-09

D9W vs Z

	RNA1-RNA1	RNA2-RNA2	RNA3-RNA3	RNA4-RNA4
**W**	0	0	0	0
***p*-value**	8.857677e-09	8.857677e-09	8.857677e-09	8.857677e-09

Supplementary Figure

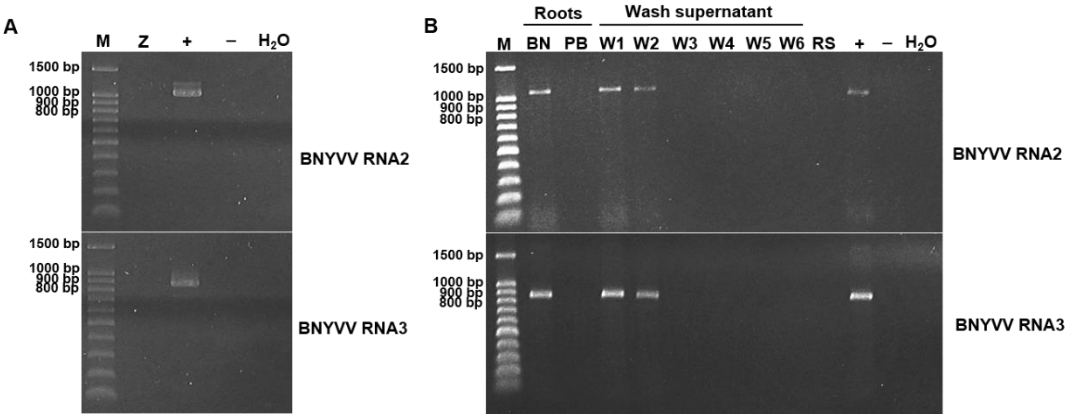

Fig. S1 Electrophoresis analysis of BNYVV RNA2 and RNA3 in *P. betae* zoospore and resting spore purifications. Z: zoospore purification; BN: BNYVV-infected *B. vulgaris* roots (without *P. betae*) that were used to validate the resting spore purification protocol; PB: *B. vulgaris* roots infested with BNYVV-free *P. betae* that were used to validate the resting spore purification protocol; W1-W6: supernatant samples after each step of the wash; RS: resting spore purification; Positive control (+): *B. vulgaris* roots infected with viruliferous *P. betae*; Negative control (-): healthy *B. vulgaris* roots.
